# bbFISH-ing in the sonication fluid

**DOI:** 10.1097/MD.0000000000016501

**Published:** 2019-07-19

**Authors:** Rares Mircea Birlutiu, Victoria Birlutiu, Razvan Silviu Cismasiu, Manuela Mihalache

**Affiliations:** a Lucian Blaga University of Sibiu, Faculty of Medicine Sibiu; FOISOR Clinical Hospital of Orthopedics, Traumatology, and Osteoarticular TB Bucharest; b Lucian Blaga University of Sibiu, Faculty of Medicine Sibiu; Academic Emergency Hospital Sibiu—Infectious Diseases Clinic, Sibiu; c Carol Davila University of Medicine and Pharmacy Bucharest, Romania; FOISOR Clinical Hospital of Orthopedics, Traumatology, and Osteoarticular TB Bucharest; d Lucian Blaga University of Sibiu, Faculty of Medicine Sibiu, Romania.

**Keywords:** bbFISH, biofilm, diagnostic, molecular diagnostic, PJI, Prosthetic joint infection, sonication

## Abstract

By 2030, the annual number of combined total hip and knee arthroplasty is estimated to reach 3.5 to 4 million in the US alone. In the context of a constant increase of the number of primary and revision total hip and knee arthroplasty, an increased risk of complication is expected. Prosthetic joint infections (PJIs) represent major cause of healthcare expenditure and morbidity. PJI still remain the most common and feared arthroplasty complication. A rapid and correct diagnosis of infection is decisive for a correct therapeutical management. In this setting, the Academic Emergency Hospital Sibiu adopted and implemented, with the beginning of September 2016, a new strategy for the diagnosis of PJIs strategy that uses sonication and beacon-based fluorescent in situ hybridization (bbFISH) technology.

Until November 2017, 40 patients (40 retrieved implants) were enrolled in the study. Sonication fluid (SF) was collected after sonication of the implants, and samples were harvested on aerobic and anaerobic culture media. A bbFISH was used as a rapid method of bacteria detection.

16 patients were diagnosed with PJIs (all 16 patients presented a positive culture of the SF). Comparing bbFISH with culture, 11 samples tested true-positive. As the kit doesn’t contain probes for *Pseudomonas fluorescens* or *Ralstonia pickettii*, 4 strains of *R pickettii* and 1 strain of *P fluorescens* that was associated with *Staphylococcus epidermidis* were not detected.

Bacteria culture of SF remains the gold standard. bbFISH holds promise to be a diagnostic tool for rapid identifying of PJIs. The bbFISH assay needs to be optimized for the detection of bacterial strains that are relevant for the PJIs field.

## 1 Introduction

Prosthetic joints are increasingly used in the growing population of the elderly, mainly for treatment of the degenerative joint disease (osteoarthritis), but also for bone fractures (femoral head fractures). By 2030, the annual number of combined total hip and knee arthroplasty is estimated to reach 3.5 to 4 million in the US alone.^[1]^ Arthroplasty surgeries have a significant effect on the quality of life, on reducing symptoms, on regaining physical function, and on improving mobility and regaining the independence of daily routines.^[2]^ In the context of a constant increase of the number of primary and revision total hip and knee arthroplasty performed each year, an increased risk of complication is expected (both prosthetic joint infection and aseptic loosening). Prosthetic joint infections occur at a frequency of 1% to 3% and represent major cause of healthcare expenditure and morbidity.^[3,4]^ Each year, in the US, over 12 million cases of infections associated with biofilm are reported, biofilm-related infections, most common being associated with orthopedic implants.^[5]^ The biofilm is a structure consisting of bacterial cells (1 or more microorganism species), an aggregate of microorganisms, in which cells are surrounded by a matrix produced by the bacteria, a structure in which the cell that are adherent to each other and/or to a surface.^[6]^ In Sibiu, Romania, according to the Romanian Arthroplasty Register in 2017, 109 primary and 10 revision surgeries of total hip arthroplasty, and 32 primary and 3 revision surgeries of total knee arthroplasty were performed. Prosthetic joint infection (PJI) still remains the most common and feared arthroplasty complication. Having in mind the fact that the management of aseptic failure is different from the one of prosthetic joint infections, an accurate diagnosis is crucial for treatment outcome, a diagnostic that still remains a difficult one.^[7]^ A rapid and correct diagnosis of infection is decisive for a correct therapeutical management. In this setting, the Academic Emergency Hospital Sibiu Romania adopted and implemented, with the beginning of September 2016, a new strategy for the diagnosis of prosthetic joint infections, strategy that uses sonication and beacon-based fluorescent in situ hybridization (bbFISH) technology.

## 2 Materials and methods

### 2.1 Study design

A single-center observational cohort ongoing study is conducted at the Academic Emergency Hospital Sibiu, Romania, a county hospital with 1054 beds. The study protocol was reviewed and approved by the institutional review board before patient inclusion. A standardized diagnostic system is applied to all patients that underwent a surgical intervention of revision of a joint prosthesis, to determine the cause of prosthesis failure. The implemented diagnostic strategy includes standardized sampling of 4 intraoperative tissue specimens (1 of the samples is used for the histopathological examination (periprosthetic membrane) and 3 of there are sent to the microbiological laboratory for bacterial cultures), sonication of removed orthopedic prosthetic components or of polymethylmethacrylate spacer and harvesting of the sonication fluid, incubation and cellular count of the synovial fluid, and the assessment of the sonication fluid using a bbFISH kit (hemoFISH Masterpanel, Miacom diagnostics GmbH Düsseldorf, Germany) as a rapid method of bacteria detection. The specimens are inoculated on aerobic and anaerobic culture media, and a 14 days period of incubation was implemented.

### 2.2 Study population

We prospectively included all consecutive patients aged over 18 years, hospitalized from September 2016 through November 2017 in our hospital, patients that underwent a joint arthroplasty revision surgery, in whom the prosthesis or part of it (such as the liner) was removed for any reason. Detailed information was abstracted from the medical records using a standardized data collection form. Medical records were evaluated for the following data: demographic characteristics; clinical, radiographic, laboratory, histopathological, and microbiological data; type of surgical management; previous antimicrobial therapy; and information about the primary arthroplasty and subsequent revisions (if any intervention was performed). The necessary information was available for all the enrolled patients. We have followed the patients until they develop a treatment failure, have died or they were loss during the follow-up period. We used descriptive statistics to summarize the demographic, clinical and treatment aspects and we analyzed the data using PSPP, version 1.0.1. The level of statistical significance was set at *P* <.05.

### 2.3 Study definitions and classification

Prosthetic joint infection was defined using criteria from the new definition for periprosthetic joint infection from the workgroup of the Musculoskeletal Infection Society published by Javad Parvizi et al:

(I) there is a sinus tract communicating with the prosthesis; or
(II) a pathogen is isolated by culture from at least 2 separate tissue or fluid samples obtained from the affected prosthetic joint; or
(III) four of the following 6 criteria exist: elevated serum erythrocyte sedimentation rate (ESR) and serum C-reactive protein (CRP) concentration; elevated synovial leukocyte count; elevated synovial neutrophil percentage (PMN%); presence of purulence in the affected joint; isolation of a microorganism in 1 culture of periprosthetic tissue or fluid, or greater than 5 neutrophils per high-power field in 5 high-power fields observed from histologic analysis of periprosthetic tissue at × 400 magnification.^[8]^

To determine whether or not there is an acute, late chronic or acute late prosthetic joint infection, we used the classification proposed by Zimmerli et al, that defines the prosthetic joint infections as early (occurring within 3 months postoperatively), delayed (3–24 months) and late (>24 months).^[3]^

### 2.4 Histopathological classification of joint implant related pathology

Intraoperatively tissue samples from of the periprosthetic membrane were taken and sent to our histopathological laboratory. We used the revised histological classification proposed by Krenn and Morawietz to assess the samples, classification that divides the neosynovium/periprosthetic membrane as:

(a) Type-I membrane: infiltrate of macrophages and multinuclear giant cells with positive macro- and micro polyethylene particles.
(b) Type-II membrane: partly diffuse, partly confluent infiltrate of CD 15 positive neutrophilic granulocytes with formation of micro abscesses (indirect immunohistochemistry, magnification 200 × ).
(c) Type-III membrane: characteristics and combination of both the Type-I membrane and Type-II membrane.
(d) Type-IV membrane: fibrous connective tissue, no abrasion particles, no detectable infiltration of inflammatory cells.
(e) Type-I or type-IV membrane with adverse reaction or particle induced arthrofibrosis.^[9]^

### 2.5 Synovial fluid and periprosthetic cultures

Synovial fluid was aspirated preoperatively. The aspirate was transferred into 2 sterile vials. One of the vials contained ethylenediaminetetraacetic acid (EDTA) for the determination of the leukocyte count and the percentage of granulocytes. The other was a native vial for bacterial culture. The synovial fluid was inoculated and incubated aerobically, anaerobically and in high concentration of CO_2_ at 37°C for 14 days and inspected daily for bacterial growth. Isolated bacteria are identified using the VITEK 2 Compact analyzer (bioMérieux, Marcy-l’Étoile, France). The minimum inhibitory concentrations (MICs) are assessed according to the European Committee on Antimicrobial Susceptibility Testing breakpoints (EUCAST). Previous reports have established optimal cutoff points for the diagnosis of bacterial prosthetic joint infections with a synovial leukocyte count greater than >1.7 G/L or >65% neutrophils in knee prosthesis or leukocyte count >4.2 G/L or >80% neutrophils in hip prosthesis.^[2,10]^

### 2.6 Sonication of the retrieved implants and sonication fluid cultures

Both prosthesis or polymethylmethacrylate spacer are retrieved and sonicated. In the operating theater, Ringer's or saline solution in added in the sterile containers. Containers that are previously sterilized according to the manufacturer and double packed. The implants are processed within 30 minutes by sonication (1 min) using an ultrasound bath (BactoSonic14.2, Bandelin GmbH, Berlin, Germany) at a frequency of 42 kHz and a power density of 0.22W/cm^2^. The resulting sonication fluid is vortexed, and 50 mL of sonication fluid is centrifuged at 2500 rpm for 5 minutes. The resulted precipitate is inoculated onto Columbia agar with sheep blood (incubated aerobically, anaerobically and in high concentration of CO_2_), Sabouraud plate, MacConkey agar plate, glucose broth, lactose broth, and thioglycollate broth. Cultures are incubated at 37°C for 14 days and inspected daily for bacterial growth. Isolated bacteria are identified using the VITEK 2 Compact analyzer (bioMérieux, Marcy-l’Étoile, France). The MICs are assessed according to the EUCAST breakpoints. The sonication fluid cultures were considered positive, if >50 CFU/mL of sonication fluid were counted.^[11,12]^

### 2.7 Periprosthetic tissue cultures

Tissue samples are collected in sterile vials and were individually homogenized in 1 mL thioglycolate broth. Tissue homogenate samples (1 mL) are inoculated onto Columbia agar with sheep blood (incubated aerobically, anaerobically and in high concentration of CO_2_), Sabouraud plate, MacConkey agar plate, glucose broth, lactose broth, and thioglycollate broth. Cultures are incubated at 37°C for 14 days and inspected daily for bacterial growth. Isolated bacteria are identified using the VITEK 2 Compact analyzer (bioMérieux, Marcy-l’Étoile, France) and the MICs are assessed according to the EUCAST breakpoints.

### 2.8 Molecular identification of bacteria by 16S rRNA bbFISH (beacon-based fluorescent in situ hybridization) technology from the sonication fluid

In addition, as a rapid method of bacteria detection, molecular identification of bacteria by 16S rRNA bbFISH (beacon-based fluorescent in situ hybridization) technology using a bbFISH kit (hemoFISH Masterpanel, miacom diagnostics GmbH Düsseldorf, Germany), was also implemented. Miacom diagnostics GmbH has combined the classical FISH technology with the usage of fluorescently labelled DNA-molecular beacons as probes, making it an easy procedure.^[13]^ The bbFISH assay is performed according to the manufacturer using a sample of resulted precipitate from 50 mL of sonication fluid that is centrifuged at 2500 rpm for 5 minutes. The kit contains beacons for the detection of *Staphylococcus* spp., *Staphylococcus aureus*, *Streptococcus* spp., *Streptococcus pneumoniae*, *Streptococcus agalactiae*, *Enterococcus faecium*, *Enterococcus faecalis*, *Enterobacteriaceae*, *Escherichia coli*, *Klebsiella pneumoniae*, *Proteus mirabilis*, *Pseudomonas aeruginosa*, *Acinetobacter* spp., and *Stenotrophomonas maltophilia.*

## 3 Results

### 3.1 Demographic characteristics of the enrolled patients

A total of 40 patients were enrolled in this study in the analyzed period, representing a total number of 40 retrieved implants, prosthesis (n=36) or polymethylmethacrylate spacers (n=4). The diagnosis of aseptic failure was in 24 cases (60%) and prosthetic joint infections in 16 cases (40%). The 16 cases of prosthetic joint infections included patients with hip (n=7), knee (n=6) prosthesis and 3 patients with a prosthetic joint infection diagnosed after the sonication of a hip polymethylmethacrylate spacers. Regarding the 24 cases of aseptic failure, we enrolled in the study patients with hip (n=13), and knee (n=10) prosthesis and 1 patient with a hip polymethylmethacrylate spacer. Regarding the polymethylmethacrylate (PMMA) sonicated spacers, the first surgeries were performed before the introduction of the diagnostic strategy, and no bacteria were isolated. The mean patient age at the time of infection was 67.9 years (range, 56–83 years), and 9 (56.25%) were male patients. The mean patient age at the time of revision of the aseptic failure cases was 65.53 years (range, 44–79 years), and 13 (54.16%) were male patients. Among the 16 prosthetic joint infections cases, 2 (12.5%) were early postoperative, 4 (25%) were delayed postoperative (low-grade) and 10 (62.5%) were late infections. Regarding the management of the 24 patients with aseptic failure, in 23 of the patients 1-step exchange procedure was performed, and in 1 case a 2-step exchange was performed due to a high suspicion of infection. The most common underlying joint disorder was osteoarthritis (n=28), followed by rheumatoid arthritis (n=7) and trauma (n=5). The mean time and the standard deviation between the primary surgical intervention and time of infection were 62 ± 56 months (range, 0.5–202 months).

### 3.2 Clinical presentation, comorbidity, and laboratory tests

The interval between implantation of the prosthesis to onset of symptoms ranged from 2 weeks to 17 years. However, 62.5% of prosthetic joint infections episodes occurred >24 months after implantation. The signs and symptoms presented by the patients with prosthetic joint infections are shown in Table 1. A sinus tract was evident in 4 cases of prosthetic joint infections. Only 3 of the 40 (7.5%) patients did not have any comorbidities, all 3 being cases of aseptic loosening. Eight (20%) patients had more them 3 comorbidities, 4 of them being cases of prosthetic joint infections. The most frequent comorbidities are presented in Table 2. Erythrocytes sedimentation rate (ESR), C-reactive protein (CRP), white blood cell (WBC), fibrinogen and total synovial fluid leukocyte count and neutrophil percentage are presented in detail in Table 3. The mean ESR values for prosthetic joint infection cases and aseptic loosening cases were 46.5 mm/hour and 23 mm/hour, respectively (*P* = .65). The mean CRP values for prosthetic joint infection cases and aseptic loosening cases were 30.5 mg/L and 15 mg/L, respectively (*P* = .122). The mean WBC count for prosthetic joint infection cases and aseptic loosening cases was 8.634∗10.3/mL and 8.234∗10.^3^/mL, respectively (*P* = .623). The mean fibrinogen level for prosthetic joint infection cases and aseptic loosening cases was 494 mg/mL and 419 g/mL, respectively (*P* = .649). The mean total synovial fluid leukocyte counts and differential cell counts for prosthetic joint infection cases was 13.08 G/L with 69% neutrophils and for aseptic loosening cases mean total synovial fluid leukocyte counts were 0.2 G/L, respectively (leukocyte count: *P* = .01, neutrophil percentage: *P* = .278).

**Table 1:**
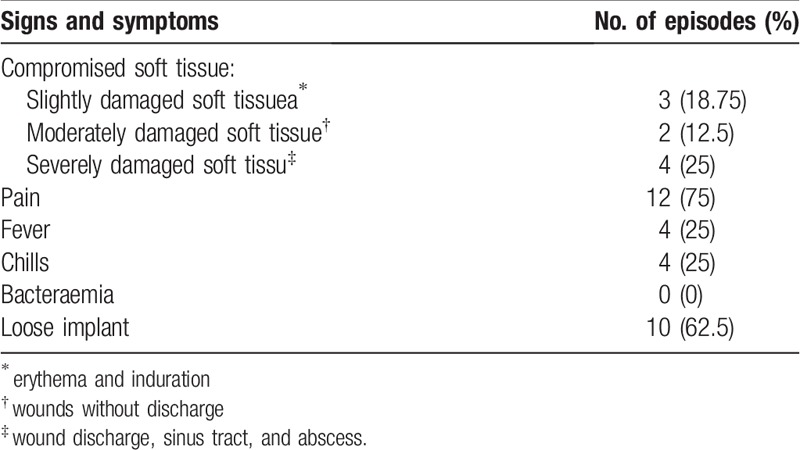
Signs and symptoms of the 16 patients with periprosthetic joint infection.

**Table 2:**
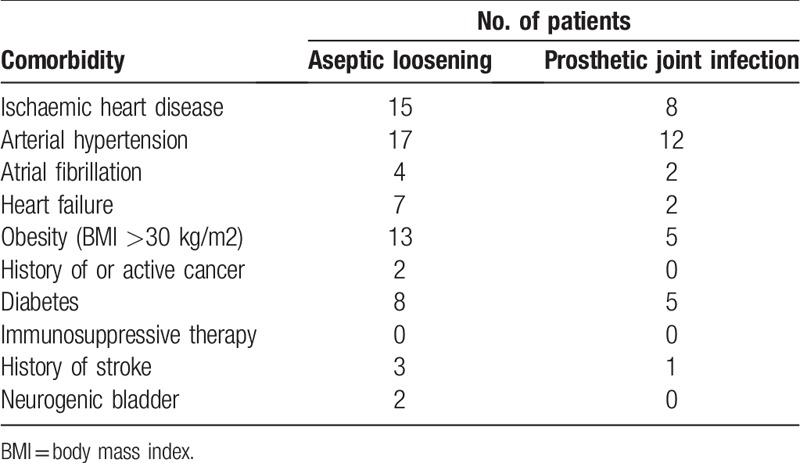
Common comorbidities of the 40 patients enrolled in the study.

**Table 3:**
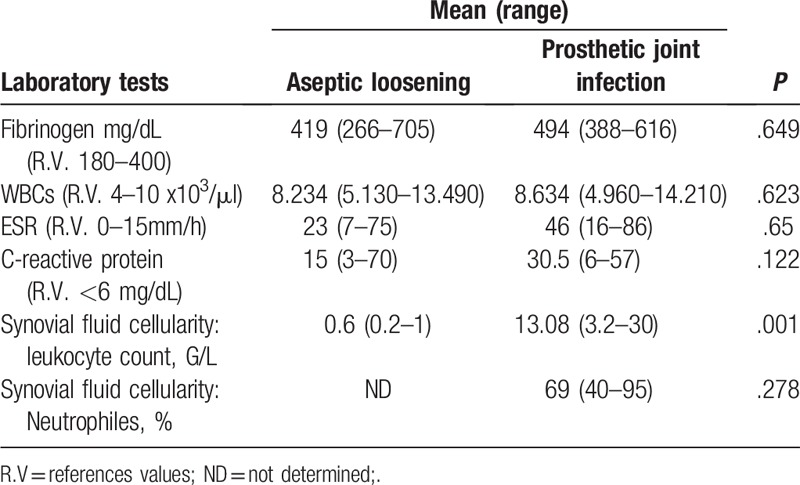
Laboratory tests for the 40 patients enrolled in the study.

### 3.3 Histopathological classification of joint implant related pathology

The assessment of the intraoperatively periprosthetic membrane of the prosthetic joint infection cases was: type-I membrane (n=2), type-II membrane (n=7), type-III membrane (n=4), type-IV membrane (n=2), and type-IV membrane with particle-induced arthrofibrosis (n=1). The classification of the periprosthetic membrane of the aseptic loosening was as follows: type-I membrane (n=6), type-IV membrane (n=12), and type-IV membrane with particle-induced arthrofibrosis (n=6).

### 3.4 Microbiologic characteristics

**Figure 1:**
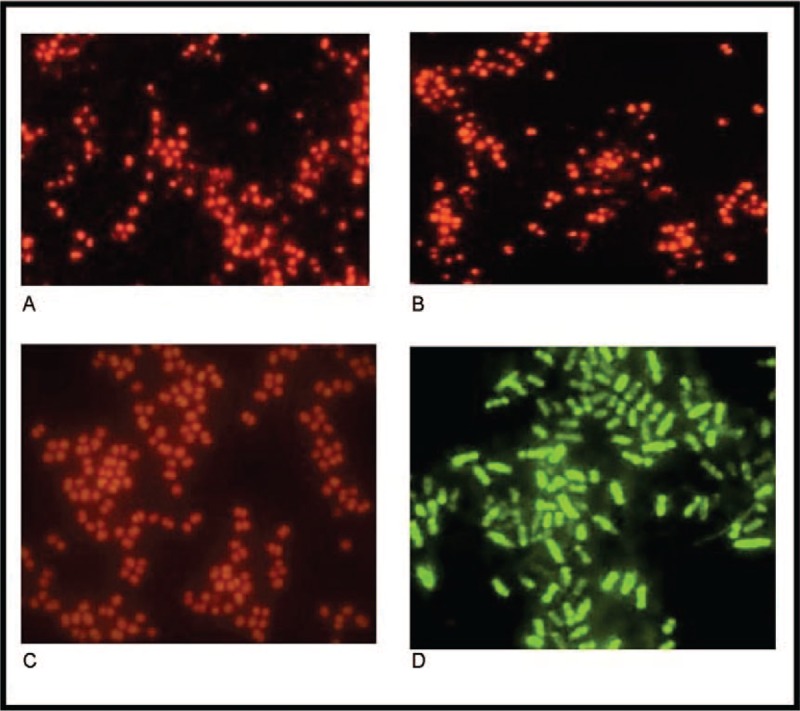
Fluorescence microscopy in immersion oil images. A and B *Staphylococcus epidermidis,* C *Enterococcus faecalis,* and D *Klebsiella* spp.

Table 4 summarizes the microbiological findings of the 16 cases of prosthetic joint infection. From the sonication fluid culture, a single causative agent was isolated in 14 (87.5%) and a polymicrobial infection in 2 (12.5%) cases. From the synovial fluid that was aspirated preoperatively, 3 strains were isolated, all of them as single causative organism. From the periprosthetic tissue cultures, a single causative agent was isolated in 7 (43.75%) and a polymicrobial infection in 3 (18.75%), in 1 case of the polymicrobial infections 1 of the strains was isolated just in 1 of the 3 samples. There were 11 strains of Gram-positive bacteria isolated, of them 2 strains of Methicillin-resistant *Staphylococcus aureus* (MLSBi -inducible resistance to clindamycin strains), 6 strains of coagulase-negative staphylococci (CoNS) and 2 strains of *Enterococcus faecalis*. Methicillin resistance was detected in 4 of 6 (66%) strains of coagulase-negative staphylococci. Both strains of MRSA were susceptible to rifampicin, and all strains of CoNS presented an intermediate susceptibility to rifampicin. 8 strains of Gram-negative bacteria isolated. 4 of the Gram-negative prosthetic joint infections were caused by *Ralstonia pickettii*. Comparing the bbFISH assay with culture, 11 samples tested true-positive. As the kit does not contain probes for *Pseudomonas fluorescens* or *R pickettii*, 4 strains of *R pickettii* and 1 strain of *P fluorescens* that was associated with *Staphylococcus epidermidis* were not detected. Details of the performance of the bbFISH assay are presented in Table 5. (Fig. 1)

**Table 4:**
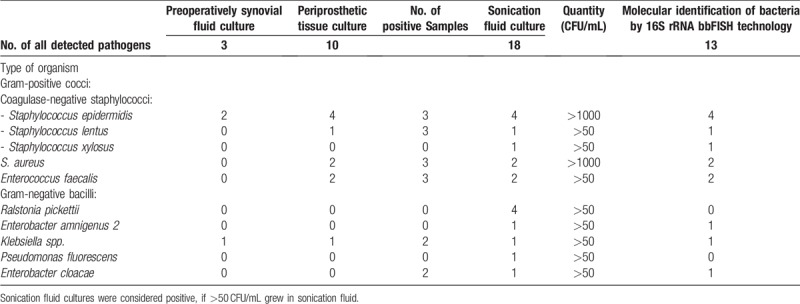
Microbiological characteristics of the 16 cases of prosthetic joint infection, taking into account the type of diagnostic specimen.

**Table 5:**
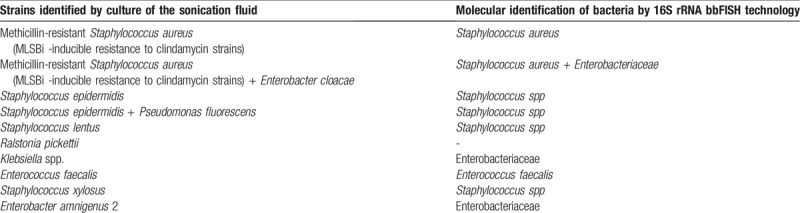
bbFISH kit identification.

### 3.5 Time to culture positivity in patients with prosthetic joint infection

After 1 day of incubation, 2 (66%) of preoperatively synovial fluid, 6 (60%) of intraoperative tissue and 5 (27%) of sonication fluid cultures were positive. After 2 days of incubation, 66% of preoperatively synovial fluid, 90 60% of intraoperative tissue and 11 (61%) of sonication fluid cultures were positive. After 3 days of incubation, 100% of preoperatively synovial fluid, 100% of intraoperative tissue and 12 (66%) of sonication fluid cultures were positive. After 4 days of incubation, all preoperatively synovial fluid, all intraoperative tissue and 13 (72%) of sonication fluid cultures were positive. After 8 days of incubation, all preoperatively synovial fluid, all intraoperative tissue and 16 (88%) of sonication fluid cultures were positive. After 12 days of incubation all cultures of the sonication fluid were positive. Prolonged incubation of intraoperative tissue and sonication fluid cultures up to 14 days did not identify additional microorganisms.

### 3.6 Comparison of diagnostic techniques

The performance of the used diagnostic methods is summarized in Table 6, table that summarizes the culture accuracy of preoperatively synovial fluid, intraoperative tissue, sonication fluid, and molecular identification of bacteria by 16S rRNA bbFISH technology, from patients with prosthetic joint infection and aseptic failure. The sensitivity of sonication fluid culture was significantly higher than that for intraoperative tissue culture and preoperatively synovial fluid (100%, 43%, and 18%, respectively). The sensitivity of the molecular identification of bacteria by 16S rRNA bbFISH technology was 68.75% (as an overall sensitivity), but if analyzing the sensitivity strictly by the bacterial agents that could be identified by the used kit, the sensitivity would be 100%.

**Table 6:**
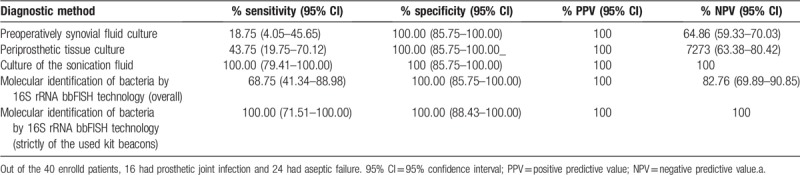
Performance of used diagnostic methods.

### 3.7 Treatment and outcome

We analyzed all 16 cases of prosthetic joint infections. Table 7 summarizes the treatment and the outcome of the enrolled patients. Surgical intervention was associated with antibiotic treatment for all patients; no patient was managed with long term suppressive antibiotic therapy. Regarding the surgical management, either 1-stage (n=9), 2-stage (n=6) or 3-stage (n=1) exchange were performed. The therapeutical management failed in 1 case due to lack of compliance for the antibiotic therapy and due to the fact that the patient was immunocompromised after renal transplant. Case that was managed with a resection arthroplasty, being a case of failed treatment for a recurrent prosthetic joint infection about the hip. A thorough debridement of all infected or devitalized tissue was done at the time of resection, the patient can bear weight and is ambulatory with an assistive device, and no reinfection at 26 months of follow up. Median duration of follow- up after revision surgery was 24 months (range 14–30). Patients who underwent a 2-stage exchange had a resection arthroplasty with placement of antibiotic (vancomycin and gentamicin) impregnated bone cement followed by delayed prosthesis re-implantation (6 weeks after prosthesis removal). Regarding the antibiotic treatment of the 2-stage exchange, 2 weeks of I.V. antibiotics + 6 weeks of p.o. followed by 6 weeks of p.o. antibiotics after the second stage. Intravenous antibiotic treatment included vancomycin in 9 episodes, linezolid in 2, levofloxacin in 2, and meropenem in 1. The most common oral antibiotic prescribed was cotrimoxazole in 15 episodes, followed by ciprofloxacin in 2 case and levofloxacin in 1 case. The duration of intravenous treatment was 2 weeks, except 1 case. Total treatment duration was 3 months, except 1 case.

**Table 7:**
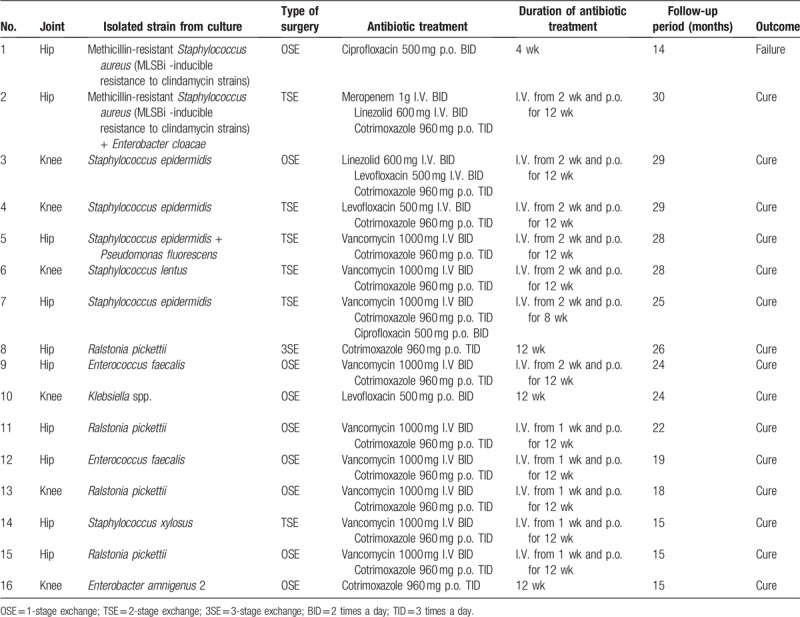
Treatment and the outcome of the enrolled patients.

## 4 Discussions

So far, there is still no reference standard for the diagnosis of prosthetic joint infection.^[14]^ A multidisciplinary approach is mandatory for the management of each case of prosthetic joint infection.^[2]^ Besides conventional microbiological methods like culture, prosthesis sonication and molecular methods are improving the diagnostic performances.^[15]^ Analysis of periprosthetic tissue biopsy or sonicate fluid samples showed a wide range of performances. Despite the absence of a reference standard, comparisons between studies remain difficult regarding the sensitivity and specificity values of periprosthetic tissue or sonication fluid culture that range from 50% to 92% and from 65% to 94%, respectively.^[16]^ Also in our study, the sensitivity of sonication fluid culture was significantly higher than that for intraoperative tissue culture and preoperatively synovial fluid (100%, 43%, and 18%, respectively).

There are many publications that have demonstrated the usefulness of “rapid methods” like broad-range PCR in diagnosing prosthetic joint infections. Studies that compared broad-range PCR with culture of sonicate fluid and reported that they have equivalent performance for prosthetic joint infection diagnosis.^[17]^

We compared preoperatively synovial fluid, intraoperative tissue, sonication fluid, and molecular identification of bacteria by 16S rRNA bbFISH technology. Our literature search revealed just 1 study that used for molecular identification of bacteria 16S rRNA bbFISH technology. Study published by Boot et al and in which they analyzed the sonication fluid that was collected by sonicating retrieved implants from 62 patients. They reported that culture resulted in 27 positive and 35 negative samples. 16S rRNA bbFISH technology resulted in 24 samples that tested true-positive and 32 samples true-negative. Interestingly 3 samples tested false-negative and 3 samples false-positive. The 3 samples that tested positive with FISH were culture negative. They also concluded that this result could indicate a higher sensitivity for detection of bacteria with FISH than with culture.^[18]^ In our study, the sensitivity of the molecular identification of bacteria by 16S rRNA bbFISH technology was 68.75% (as an overall sensitivity), but if analyzing the sensitivity strictly by the bacterial agents that could be identified by the used kit, the sensitivity would be 100%. The sonication procedure followed by 16S rRNA bbFISH was positive in 68.72% of studied cases, using the assessed 16S rRNA bbFISH kit. Beside the identification of etiological agents, the assay could also identify contaminating agents that is possible due to the manipulation of the sonication containers. 16S rRNA bbFISH technology is a fast assay that provides results in 30 minutes.

In our study from the synovial fluid that was aspirated preoperatively, 3 strains were isolated. Preoperatively, the causative organism was known just in 3 cases, compared with other studies in which in >80% of cases, the infecting agent and its susceptibility were known before surgery.^[19]^

Interestingly, we were able to identify 4 cases (1 case being already published) of prosthetic joint infections caused by *R pickettii*, all 4 strains being isolated from the cultures of the sonication fluid. *R pickettii*, is a Gram-negative, nonfermentative, oxidase-positive bacteria. It is an opportunistic pathogen associated with nosocomial infections due to contamination of sterile water, saline solution, disinfectants, blood culture bottles, and venous catheters. Exceptionally being isolated from the mouth and upper respiratory tract, being responsible for lung abscess after necrotizing pneumonia in elderly, non-hospitalized patients or lobar pneumonia associated with severe respiratory insufficiency. *R pickettii* bacteremia, was associated also with the use of contaminated antiseptic solutions—chlorhexidine solutions, and distilled water or with extracorporeal membrane oxygenation, and with the contamination of irrigation system in obstetric care. *R pickettii* is extremely rare associated with prosthetic joint infections. From our previously experience in the management of a prosthetic joint infection with *R pickettii*, and from the antibiotic sensitivity, of the 3 last isolated strains, at trimethoprim/sulfamethoxazole, cotrimoxazole 960 mg p.o. TID seems to be a way of managing this cases. No failure of treatment at a mean follow up of 9 months.^[20]^

The main limitations of our study are the small sample size and the short period of observation. Larger studies are needed to confirm these results. Nevertheless, our results are very promising.

## 5 Conclusions

In conclusion, bacteria culture of sonication fluid remains the gold standard in diagnosing prosthetic joint infections. Negative culture of preoperative joint aspiration and soft tissues surrounding the implant and periprosthetic interface membrane obtained intraoperatively, do not exclude the presence of bacteria on the implants. The 16S rRNA bbFISH is a new successful molecular assay, supplementing traditional approaches, and speeding up the diagnosis. The 16S rRNA bbFISH assay needs to be optimized for the detection of bacterial strains that are relevant for the prosthetic joint infections field like *Cutibacterium* (formerly *Propionibacterium*) *acnes* and why not *R pickettii* or *Pseudomonas* spp.

## Author contributions

**Conceptualization:** Rares Mircea Birlutiu, Victoria Birlutiu.

**Data curation:** Rares Mircea Birlutiu, Victoria Birlutiu.

**Formal analysis:** Rares Mircea Birlutiu, Victoria Birlutiu, Razvan Silviu Cismasiu, Manuela Mihalache.

**Investigation:** Rares Mircea Birlutiu, Victoria Birlutiu.

**Methodology:** Rares Mircea Birlutiu, Victoria Birlutiu.

**Project administration:** Rares Mircea Birlutiu, Victoria Birlutiu.

**Resources:** Rares Mircea Birlutiu, Victoria Birlutiu, Razvan Silviu Cismasiu.

**Supervision:** Rares Mircea Birlutiu, Victoria Birlutiu.

**Validation:** Rares Mircea Birlutiu, Victoria Birlutiu, Razvan Silviu Cismasiu, Manuela Mihalache.

**Visualization:** Rares Mircea Birlutiu, Victoria Birlutiu, Razvan Silviu Cismasiu, Manuela Mihalache.

**Writing – original draft:** Rares Mircea Birlutiu, Victoria Birlutiu, Razvan Silviu Cismasiu.

**Writing – review & editing:** Rares Mircea Birlutiu, Victoria Birlutiu.
